# Growth and Liquid-Phase Exfoliation of GaSe_1−x_S_x_ Crystals

**DOI:** 10.3390/ma15207080

**Published:** 2022-10-12

**Authors:** Madi Aitzhanov, Nazim Guseinov, Renata Nemkayeva, Yerulan Sagidolda, Zhandos Tolepov, Oleg Prikhodko, Yerzhan Mukhametkarimov

**Affiliations:** National Nanotechnological Laboratory, al-Farabi Kazakh National University, 71 Al-Farabi Ave., Almaty 050040, Kazakhstan

**Keywords:** layered gallium selenide/sulfide crystals, stoichiometric melt, liquid-phase exfoliation

## Abstract

In recent years, interest in the liquid-phase exfoliation (LPE) of layered crystals has been growing due to the efficiency and scalability of the method, as well as the wide range of practical applications of the obtained dispersions based on two-dimensional flakes. In this paper, we present a comparative study of as-grown and liquid-phase exfoliated GaSe_1−x_S_x_ flakes. Bulk GaSe_1−x_S_x_ crystals with x ~ 0, 0.25, 0.5, 0.75, 1 were synthesized by melting stoichiometric amounts of gallium, selenium, and sulfur particles in evacuated ampoules. X-ray diffraction analysis showed that the crystal structure does not change considerably after LPE, while the analysis of the Raman spectra revealed that, after liquid-phase processing in IPA, an additional peak associated with amorphous selenium is observed in selenium-rich GaSeS compounds. Nevertheless, the direct and indirect transition energies determined from the Kubelka-Munk function for LPE crystals correlate with the band gap of the as-grown bulk GaSeS crystals. This finding is also confirmed by comparison with the data on the positions of the photoluminescence peak.

## 1. Introduction

Today, two-dimensional (2D) materials attract tremendous research interest due to the promising properties that they exhibit after exfoliation from the bulk form. Recently, the method of liquid-phase exfoliation (LPE) of layered crystals is becoming more and more popular since liquids with 2D flakes can be easily deposited on any surface using printers or spray coating devices [1,2,3,4]. Various 2D nanomaterials, such as graphene, transition metal dichalcogenides (TMDs), and black phosphorus (BP) have been obtained via this method. It is reported that the advantages of devices based on liquid-exfoliated Van der Waals crystals include not only cost-effectiveness and scalability but also high flexibility, which facilitates their application in wearable electronics [5].

Recently, the properties of LPE dispersions of gallium selenide (GaSe) and gallium sulfide (GaS) have been investigated and some of their photoelectrochemical (PEC) applications have been demonstrated [6,7,8,9].

It is known that 2D GaSe layers are active in visible light, while GaS flakes are more active in the UV region due to the energy band gap, which is ~2 eV and ~2.58 eV for GaSe and GaS crystals, respectively. According to the data in the literature, GaSe mainly exhibits p-type conductivity, while GaS is an n-type semiconductor [10,11,12]. It is interesting that the corresponding transition ternary crystals also have a layered structure with predominantly n-type conductivity [13,14]. Depending on the Se/S ratio, the band gap of GaSeS compounds varies between that of GaSe and GaSe. All of these crystals demonstrate the same structure with Y-Ga-Ga-Y alternation, where Y = S or Se. Depending on the stacking of layers, different polytypes (α, β, ε, and δ) can exist. Similar to the other two-dimensional (2D) Van der Waals nanomaterials, GaSeS crystals exhibit thickness-dependent electronic properties that become significant in the case of a few numbers of layers.

Usually, GaSe and GaS crystals along with their ternary compounds are synthesized by the Bridgman-Stockbarger (BS) method [15,16,17,18,19]. In this technique, the growth of a single crystal is carried out in a two-zone furnace with a sharp temperature gradient. Certainly, this method gives the opportunity to obtain various compositions of GaSe_1−x_S_x_ crystals with a controlled crystal quality, suitable for nonlinear optical applications [20,21]. Nevertheless, the use of GaSeS crystals grown by the BS method for LPE is not rational from an economic point of view because of the lengthy and rather expensive synthesis process.

It is important to note that, despite all the above-mentioned advantages of the method of liquid-phase exfoliation, the properties of the resultant LPE crystals strongly depend on parameters such as the type of sonication (bath or tip) and especially the liquid media. For example, Kang et al. reported that the LPE InSe film prepared using an ethanol-water mixture demonstrated several orders higher conductivity compared to the film based on InSe exfoliated in the liquid media of sodium dodecylsulfate–water [22]. Moreover, a photodetector formed by the spray-coating of InSe exfoliated in isopropanol [23] exhibited the highest broad-range photoresponsivity among similar devices. However, comprehensive studies of such LPE monochalcogenides as GaSe, GaS, and, especially, GaSe_1−x_S_x_ ternary compounds are still few.

In this work, we have chosen a simpler and faster way of synthesizing layered GaSe_1−x_S_x_ crystals, which consists of melting stoichiometric amounts of Ga, Se, and S particles in an evacuated ampoule using a single-zone furnace with a controlled temperature change. This method is often used for the preliminary preparation of a polycrystalline alloy for BS technology and is known as the first stage of the BS synthesis technique. The initial as-grown crystals were characterized by Raman spectroscopy and X-ray diffraction analysis. Next, the crystals were exfoliated via the liquid-phase method in an ultrasonic bath using isopropanol and characterized by standard measurement techniques to identify possible changes in properties after the LPE treatment.

## 2. Materials and Methods

### 2.1. GaSe_1−x_S_x_ Crystal Growth

The layered GaSe_1−x_S_x_ crystals (where x = 0, 0.25, 0.50, 0.75, 1) were grown by the stoichiometric melting of mixed particles of high-purity gallium (Ga), selenium (Se), and sulfur (S) inserted into an evacuated ampoule. Melting was carried out in the single-zone vertical furnace with temperature monitoring systems. For the production of GaSe crystals, an ampoule with Ga and Se particles was heated up to 980 °C for 30 minutes. Then the ampoule with the molten material was cooled at the rate of 2–3 °C/min to 550 °C, and the subsequent further cooling was natural. The GaS and ternary GaSe_1−x_S_x_ crystals were grown under the same conditions but at a higher temperature of 1040 °C.

### 2.2. Characterization of GaSe_1−x_S_x_ Crystals

The study of the elemental composition and microstructure of GaSe_1−x_S_x_ crystals was carried out by the method of energy dispersive analysis (EDS) on a Quanta 3D 200i scanning electron microscope (SEM). The structure and photoluminescence spectra of the obtained crystals were investigated using X-ray diffraction analysis (Rigaku) with a CuKα monochromator and Raman spectroscopy (NT-MDT) with a 473 nm laser excitation source. Diffuse reflection measurements were carried out on a Shimadzu UV-3600 spectrophotometer. The thicknesses of the LPE GaSeS flakes were measured by atomic force microscopy and SEM.

### 2.3. Photoconductivity Measurements

To study the spectral dependences of photoconductivity, planar photodetectors were created based on micromechanically exfoliated thick (~300–350 nm) GaSe_1-x_S_x_ flakes transferred onto the pre-deposited Au electrodes. The distance between electrodes was 50 µm and was the same for all prepared samples. The spectral dependence of the photoconductivity was measured using a monochromator with a 1200 mesh diffraction grating with a step of ~5 nm, and a 300 W tungsten filament lamp was used as a light source. The intensity of monochromatic light was calibrated using a UV-enhanced diode-type power meter Newport 818-UV (Newport Corporation, Irvine, CA, USA). The photoconductivity was measured at a constant voltage of 1 V for all samples. The photocurrent value was recorded using a Keithley 6485 picoammeter (Cleveland, OH, USA).

### 2.4. Exfoliation of GaSe_1-x_S_x_ Crystals

The liquid-phase exfoliation of GaSe_1−x_S_x_ crystals was carried out in anhydrous 2-propanol (IPA, Sigma Aldrich, St. Louis, MO, USA, 99.8%). To obtain a dispersion with a concentration of 1 mg/mL, 10 mg of GaSe_1−x_S_x_ crystals were mixed with 10 ml IPA and underwent sonication for 2 hours with a power of 150 W and a frequency of 35 kHz.

## 3. Results

### 3.1. Characterization of As-Grown GaSe_1−x_S_x_ Crystals

**Figure 1:**
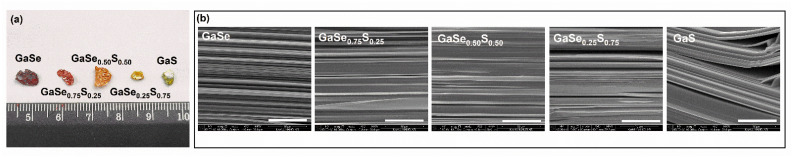
Optical (**a**) and SEM (**b**) images of the synthesized GaSe_1−x_S_x_ crystals (scale bar 10 μm).

**Figure 2:**
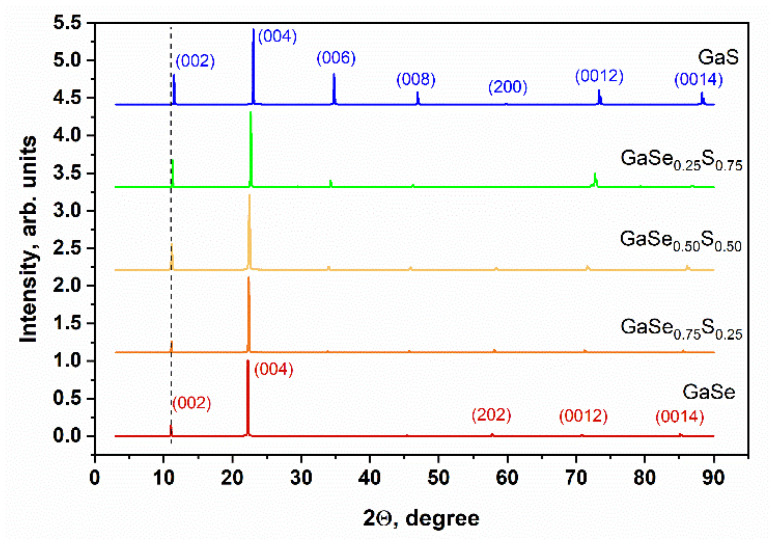
X-ray diffraction (XRD) patterns of the as-grown GaSe_1−x_S_x_ crystals.

The optical image presented in Figure 1a shows that the synthesized GaSe_1−x_S_x_ crystals (where x = 0, 0.25, 0.50, 0.75, and 1) have corresponding colors that are typical and inherent to them. SEM studies of the synthesized samples confirmed the layered structure of the GaSe_1−x_S_x_ crystals. The SEM images of the cross-sections of the crystals are illustrated in Figure 1b. The composition of the GaSe_1−x_S_x_ crystals, according to the EDS analysis, indicates their good stoichiometry. The data of the average elemental composition of GaSe_1−x_S_x_ crystals are represented in Table 1. To confirm the crystal quality of the GaSe_1−x_S_x_ crystals, X-ray diffraction analysis was carried out. The XRD pattern of the GaSe crystal shown in Figure 2 corresponds to a hexagonal structure with lattice parameters a = b = 3.76 Å, c = 15.94 Å. This crystal structure belongs to the P63/mmc space group. The analysis of the XRD pattern of the GaS crystal also indicates its hexagonal structure, but with smaller lattice parameters: a = b = 3.65 Å and c = 15.44 Å. In the case of ternary GaSe_1−x_S_x_ compounds, the lattice parameters decrease with increasing sulfur content. The determined values of the d-spacing corresponding to the (004) plane are given in Table 1. Peak analysis was carried out in accordance with the powder diffraction file with cards 01-089-2885 and 01-074-0227 for GaSe and GaS, respectively.

**Figure 3:**
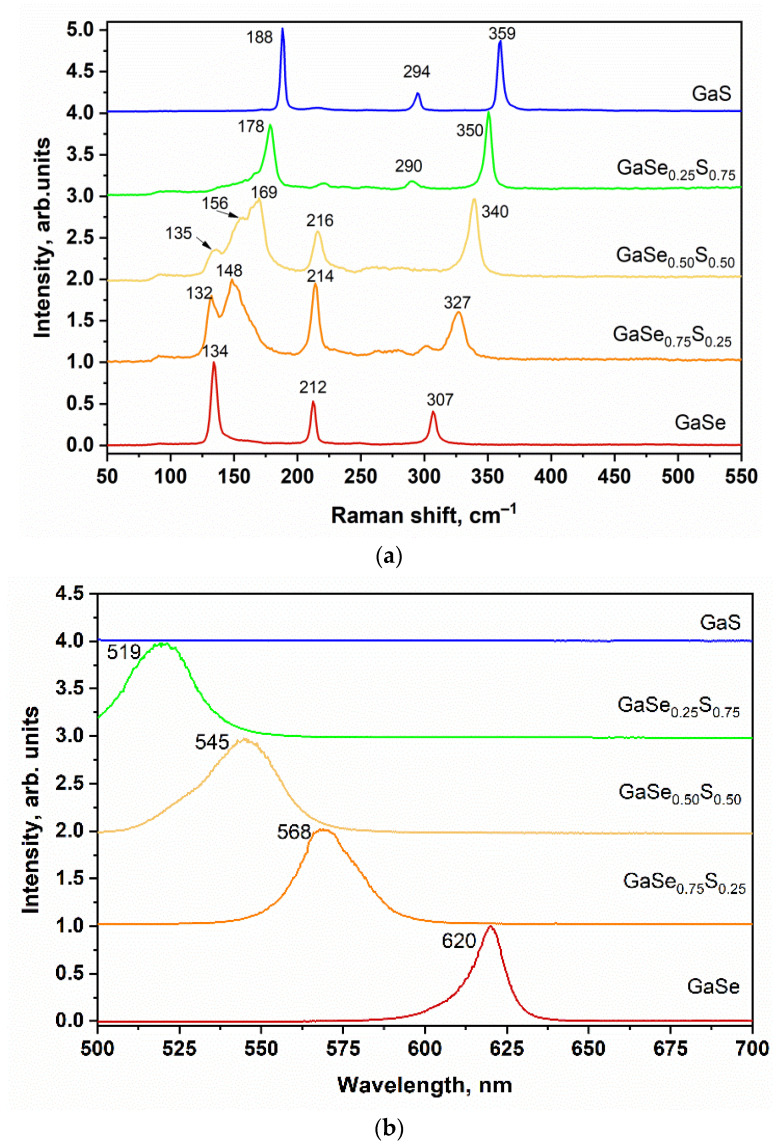
Raman (**a**) and PL (**b**) spectra of the as-grown GaSe_1−x_S_x_ crystals.

Figure 3a represents the Raman spectra of GaSe_1−x_S_x_ crystals. The Raman spectrum of the exfoliated GaSe flakes has three typical peaks at 134, 212, and 307 cm^−1^, which correspond to the A^1^_g1_, E_2g,_ and A^1^_g2_ vibrational modes, respectively. In the case of GaS, the Raman spectrum also shows the three dominant peaks at 188, 294, and 359 cm^−1^ corresponding to the same vibrational modes. Modes A^1^_g1_ and A^1^_g2_ refer to the compression/stretching out-of-plane vibrations of atoms, and E_2g_ corresponds to shear modes of in-plane vibrations. The ternary GaSe_1−x_S_x_ crystals demonstrate Raman peaks that shifted proportionally to the sulfur content and can be considered as a superposition of the corresponding GaSe and GaS Raman bands. Thus, according to the structure of ternary alloys, the three-component GaSe_0_._50_S_0_._50_ crystal demonstrates the two main peaks corresponding approximately to the average value of the Raman bands of GaSe and GaS: (134 + 188)/2 = 161 (calculated) versus 169 cm^−1^ experimental and (307 + 360)/2 = 333.5 (calculated) versus 340 cm^−1^ experimental. The observed discrepancy between the experimental and the calculated average values may indicate a slight predominance of sulfur or selenium concentration in the GaSe_1−x_S_x_ composition. At the same time, the first peak at 135 cm^−1^ in the case of GaSe_0.50_S_0.50_ crystal indicates the presence of the initial GaSe structure, while the peak at 156 cm^−1^ can be attributed to Ga_2_Se_3_, which can appear as a transition phase due to the doping process, and/or to elemental sulfur with a typical Raman peak at 153–154 cm^−1^, which can be partly intercalated between GaSeS layers. It is assumed that the peak at 216 cm^−1^ represents a slight deformation of the initial GaSe structure which leads to a shift of E_2g_ mode to 212 cm^−1^, and this peak disappears with a further increase in the sulfur content.

In contrast to TMDs such as WS_2_ and MoS_2_, which exhibit an intense PL signal only at monolayer or several layers, GaSe and Se-rich compounds emit light efficiently at the bulk state due to the pseudo-direct optical band gap, and there is a dramatic decrease in PL intensity at small thicknesses due to the transition to an indirect band gap for monolayers [24,25]. There are also several reports claiming that the observed PL signal in GaSe is close to the energy band gap of the crystal, but it emerges due to the defects induced by a slightly broken stoichiometric ratio. In addition, there are some articles that explain the quenching of PL in thin layers of GaSe by non-radiative processes associated with the surface states [26].

In this work, the PL spectra of as-grown GaSe_1−x_S_x_ crystals were measured for bulk crystals with thicknesses not less than 300 nm at room temperature. As shown in Figure 3b, all crystals except GaS demonstrate a luminescent peak that blueshifts from 620 nm for GaSe to 519 nm for GaSe_0_._25_S_0_._75_ as S content increases. It should be noted that, according to the data in the literature, a layered GaS crystal demonstrates a PL peak at 485–490 nm. However, we are unable to observe it due to device limitations [27].

**Figure 4:**
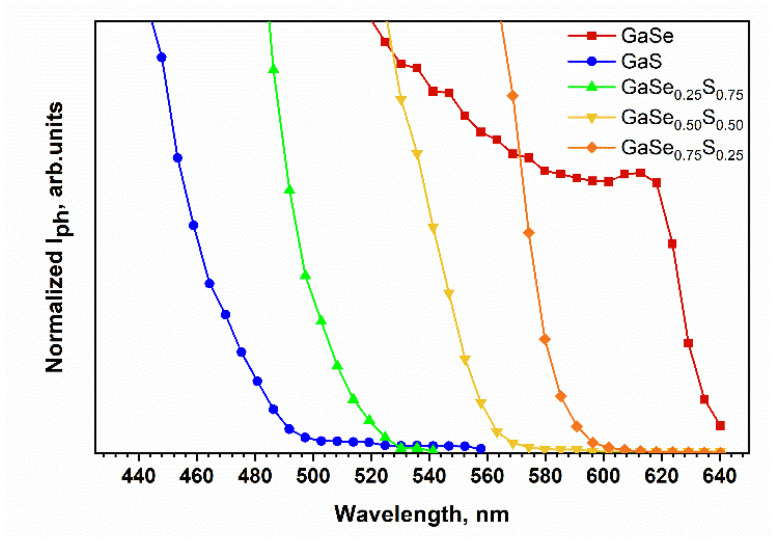
Spectral dependences of the photocurrent of the as-grown GaSe_1−x_S_x_ flakes.

As mentioned in the introductory section, the energy band gap of the ternary GaSe_1-x_S_x_ crystals depends on the sulfur/selenium ratio in the mixture. To verify this, the spectral dependence of the photoresponsivity of the GaSe_1−x_S_x_ crystals was studied. Figure 4 shows the spectral dependences of the normalized photocurrent of Au/GaSe_1−x_S_x_/Au planar structures. As can be seen, the sensitivity of all produced photodetectors increases with increasing photon energy. This observation confirms the possibility of using GaSe_1−x_S_x_ crystals for photodetector production. The photon energy, determined by the exact linear interpolation of these curves, shows that the energy gap for GaSe crystals is 1.94 eV and for GaS crystals is about 2.52 eV. For the ternary GaSe_1−x_S_x_ crystals, the determined photon energy is 2.13, 2.22, and 2.38 eV for GaSe_0.75_S_0.25_, GaSe_0.50_S_0.50,_ and GaSe_0.25_S_0.75_ crystals, respectively. The obtained values of the photon energy are close to the values of band gap reported in the literature for the corresponding crystals and correlate with the observed positions of the PL peaks, which are shown in Figure 3b.

### 3.2. Characterization of Liquid-Phase Exfoliated GaSe_1−x_S_x_ Flakes

**Figure 5:**
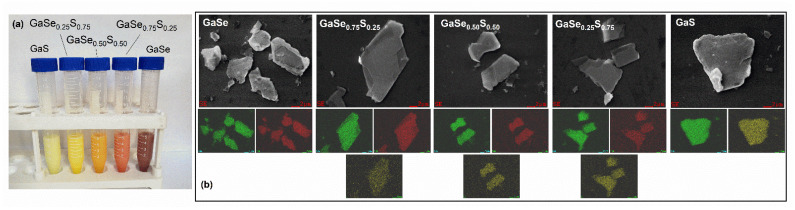
(**a**) Optical images of the GaSe_1-x_S_x_/IPA solutions; (**b**) Results of EDS mapping of the LPE GaSe_1−x_S_x_ flakes.

After the characterization of the as-grown bulk GaSe_1−x_S_x_ compounds, small amounts of all the crystals were exfoliated in anhydrous isopropanol (IPA) solvent using an ultrasonic bath. The optical images of the prepared IPA solutions with dispersed GaSe, GaS, and ternary GaSe_1−x_S_x_ crystals flakes (1 mg/mL) are illustrated in Figure 5a. These solutions contain flakes with a wide range of crystal sizes. The average lateral size of flakes is a few µm^2^, and the thickness of flakes varies from ~5 nm to ~500 nm. The results of the statistical analysis are presented in the Supplementary Materials. To check the quality of the obtained crystals after liquid-phase exfoliation, they were also investigated by SEM (and EDS analysis) measurement, XRD analysis, and Raman spectroscopy. Figure 5b shows SEM images of typical LPE GaSe_1−x_S_x_ flakes. The EDS mapping represented in Figure 5b indicates that the elemental composition of GaSe_1−x_S_x_ flakes is not significantly changed after LPE and is close to the initial values represented in Table 1.

**Figure 6:**
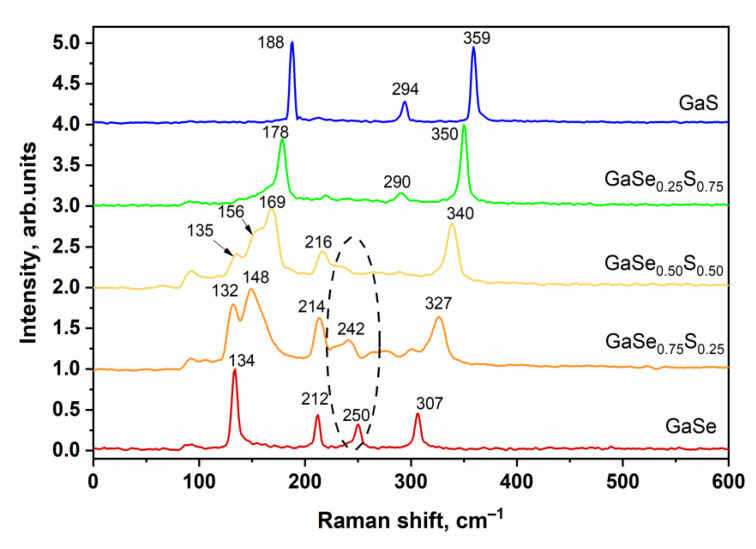
Raman spectra of the LPE GaSe_1−x_S_x_ flakes.

Figure 6 shows the Raman spectra of the LPE GaSe, GaS, and GaSeS ternary compositions. As can be seen, there are no significant changes in the structure of crystals after exfoliation. In the Raman spectra of selenium-rich GaSe and GaSe_0.75_S_0.25_ flakes, an additional peak appears at 250 cm^−1^ and 242 cm^−1^, respectively, which apparently corresponds to amorphous selenium. It should be noted that, according to the Raman spectra, the crystal structure of individual flakes remains unchanged with increasing sulfur content. The photoluminescence spectra of the same flakes do not show any noticeable changes in the PL peak position.

**Figure 7:**
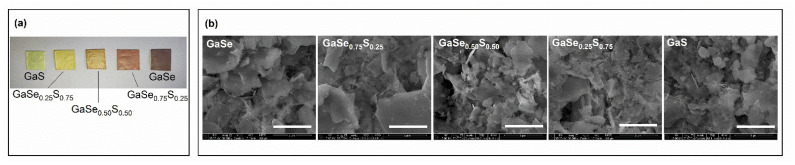
Optical (**a**) and SEM (**b**) images of the prepared LPE GaSe_1−x_S_x_ films (scale bar 5 μm).

Further analysis was carried out using films based on LPE GaSe_1−x_S_x_ flakes deposited by the drop method onto the surface of graphitic paper in an amount of 30 mg/cm^2^ (see Figure 7a). SEM images of the film surface presented in Figure 7b show that all films have randomly-arranged flakes with a wide range of sizes and shapes.

**Figure 8:**
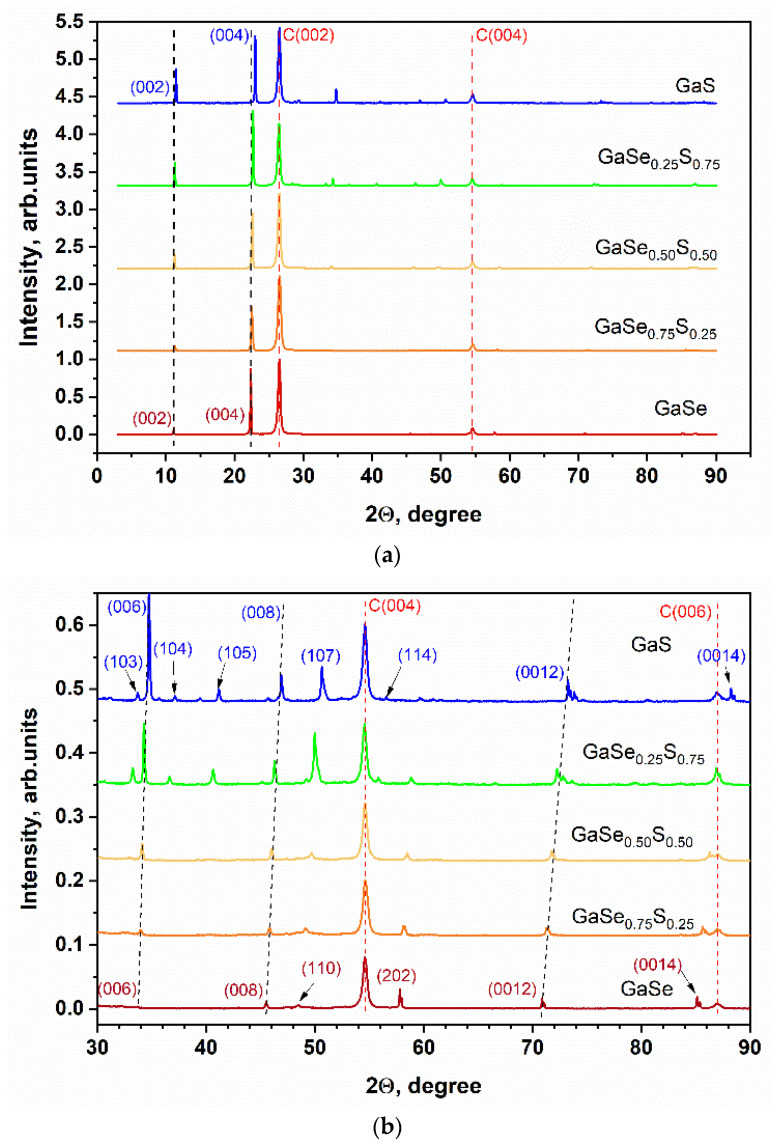
Full range (**a**) and 30–90 degrees range (**b**) XRD patterns of the LPE GaSe_1−x_S_x_ films.

The XRD analysis of GaSe_1−x_S_x_ films shows that the LPE crystals also have a predominantly layered structure. The full-range XRD pattern represented in Figure 8 indicates the presence of peaks typical for GaSe/S layered crystals that correspond to the (00x) planes, x = 2, 4, 6 … As can be seen from the figure, an increase in the sulfur content in the GaSe_1−x_S_x_ films leads to a slight shift of the peak corresponding to the (004) plane, which correlates with the XRD results of the as-grown crystals (see Figure 2). The XRD patterns in the range of angles from 30 to 90 degrees are shown in Figure 8b.

**Figure 9:**
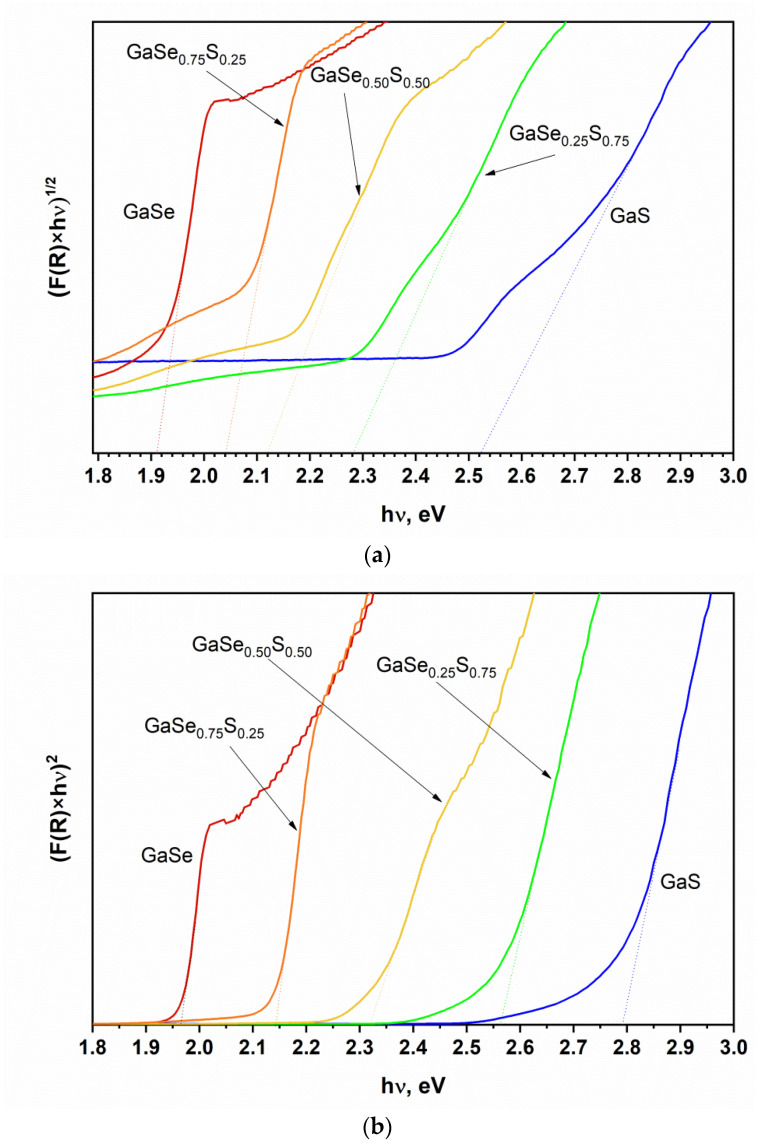
K-M plots of GaSe_1−x_S_x_ films. (**a**) indirect transition ((F(R)hν)^1/2^ vs. hν) and (**b**) direct transition energy ((F(R)hν)^2^ vs. hν).

Figure 9 shows the result of the determination of the energy band gap of GaSe_1−x_S_x_ films by the Kubelka-Munk (K-M) method from diffuse reflection spectra. The linear interpolation of the plots of (F(R)×hν)^2^ vs. hν for the quadratic approximation shows that direct transition photon energy is ~1.97 eV for GaSe and ~2.79 eV for GaS LPE films. For the films of ternary GaSeS compounds, this value is equal to ~2.14, ~2.32, and ~2.56 eV for GaSe_0.75_S_0.25_, GaSe_0.50_S_0.50,_ and GaSe_0.25_S_0.75_, respectively. The defined values of the energy of direct transition are consistent with the data in the literature for the corresponding GaSe_1−x_S_x_ crystals, as reported in [27]. The energy of the indirect transition has lower values. The determined photon energy is ~1.92 eV for GaSe films and ~2.52 eV for GaS films. Analogous to the trends in direct transition energy, the value of the energy of indirect transitions for the GaSeS ternary films increases with the increasing sulfur content and equals ~2.04, ~2.12, and ~2.28 eV for the GaSe_0.75_S_0.25_, GaSe_0.50_S_0.50,_ and GaSe_0.25_S_0.75_ compounds, respectively.

**Figure 10:**
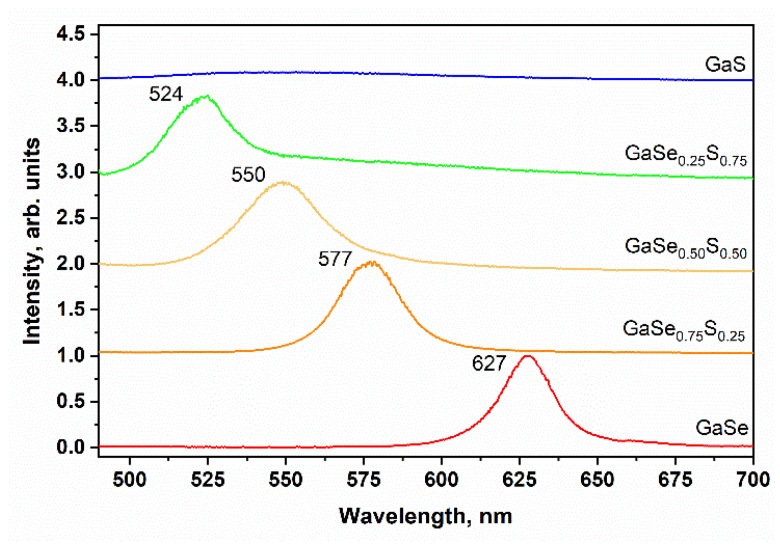
Photoluminescence spectra of the LPE GaSe_1−x_S_x_ films recorded at room temperature.

**Figure 11:**
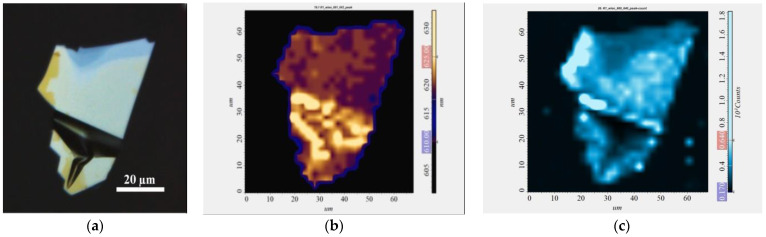
PL mapping of the GaSe flake (**a**) optical image; (**b**) map of PL peak position; (**c**) map of PL peak intensity.

The PL spectra of the LPE GaSe_1−x_S_x_ films recorded in the moving scanning regime and shown in Figure 10 demonstrate the redshift of the peaks by ~5–10 nm from the corresponding positions for as-grown crystals. This can be explained by the complex effect of the different sizes and alignments (orientations) of the flakes, as well as defects at the edges and the bending of the flakes in the films, which, in turn, can affect the integrated properties of the material. In order to demonstrate this statement, changes in the position and intensity of the PL peak for a bended GaSe flake are shown in Figure 11. One can see that the flat areas of the flake have a lower wavelength value of the PL peak position in contrast with the bended areas with the redshifted PL signal, which correlates with the trend in the PL spectra of the LPE GaSe_1−x_S_x_ films.

**Figure 12:**
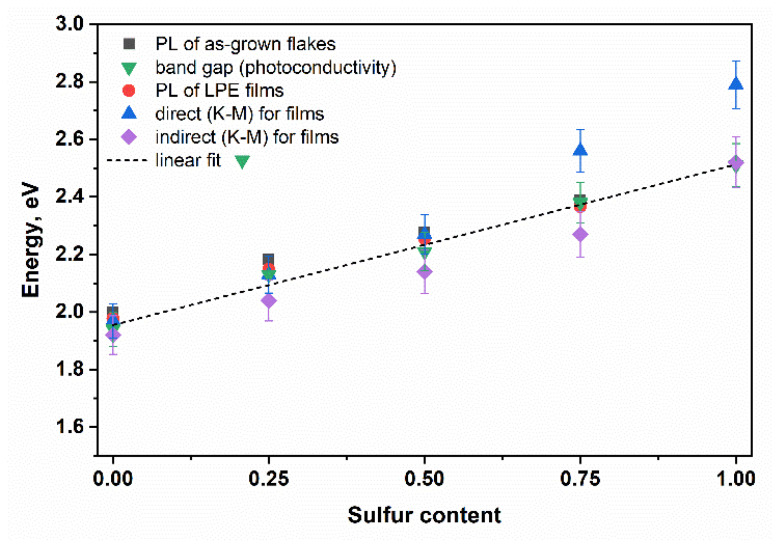
Dependence of band gap values of GaSe_1−x_S_x_ determined by different methods on sulfur content.

Figure 12 shows the values of the energy of the PL peak positions and the energies of direct and indirect transitions, determined from the interpolation of the Tauc plots of the K-M function. As can be seen in the case of selenium-rich films, i.e., GaSe and GaSe_0.75_S_0.25_, the PL peak positions and the corresponding transition energy exactly coincide with the energies of direct K-M transition. As sulfur content increases, a strong deviation from the quadratic law is observed, and these values began to differ. On the contrary, the energy band gap of the GaS flakes is successfully described by indirect transition energy. The energy band gap of GaS flakes, determined from the linear interpolation of the photoconductivity spectra, and the indirect transition energy of the films are the same. The energy values for the GaSe_0.5__0_S_0.5__0_ and GaSe_0.25_S_0.75_ films are generally in the range of errors.

According to the rules of the ternary alloys, GaSe_1−x_S_x_ crystals are expected to demonstrate linear changes in the parameters such as energy band gap, lattice parameters, and the PL peak position. However, in this contribution, the slight deviation from the linear law could be due to the possible lack of the stoichiometry associated with the non-uniform distribution of the sulfur/selenium in the GaSe/GaS host crystals. Nevertheless, GaSe_1−x_S_x_ crystals maintain their main semiconducting properties, which makes them promising for various practical applications. To confirm this, we have studied the photoresponsivity of a two-electrode photoelectrochemical cell with working electrodes based on the LPE GaSe_1-x_S_x_ films. These results are presented in the  Supplementary Materials.

It is important to note that all LPE crystals tend to degrade in IPA after a long period of time. In the Raman spectra of the flakes being dissolved in IPA for a half year, additional peaks of the amorphous selenium and sulfur have been observed. It is known that the appearance of elemental selenium and sulfur in GaSe_1−x_S_x_ crystals is usually accompanied by the formation of Ga_2_O_3_ [28].

## 4. Conclusions

In this work, the properties of the GaSe, GaS, and ternary GaSeS crystals, obtained by the simple melting of stoichiometric amounts of the Ga, Se, and S particles in a vacuum, were systematically studied. From the analysis of XRD patterns, it was found that all the synthesized crystals have a layered hexagonal structure. The structure of GaSe_1−x_S_x_ crystals did not change sufficiently after liquid-phase exfoliation in IPA, which is confirmed by the analysis of Raman spectra and XRD patterns. In addition, a comparative analysis of the band gap values determined by different methods shows that the GaSe_1−x_S_x_ LPE films demonstrate properties that are rather close to those of initial as-grown crystals.

## Figures and Tables

**Table 1:** Elemental composition and the d_(004)_—spacing of GaSe_1−x_S_x_ crystals.

Crystal Name	Ga, at.%	Se, at.%	S, at.%	d_(004)_—Spacing, Å
GaSe	49.9	50.1	-	3.98
GaSe_0_._75_S_0_._25_	49.7	38.7	11.6	3.95
GaSe_0_._50_S_0_._50_	50.1	25.2	24.7	3.93
GaSe_0_._25_S_0_._75_	49.5	12.1	38.4	3.91
GaS	49.1	-	50.9	3.87

## Data Availability

Not applicable.

## Supplementary Materials

The following supporting information can be downloaded at: https://www.mdpi.com/article/10.3390/ma15207080/s1. Figure S1. Typical (a) and contrast-adjusted (b) SEM images of the GaSeS flakes (a,b); The lateral size distribution (c–g) of the LPE GaSe1-xSx flakes. Figure S2. Typical AFM image of the GaSeS flakes (a) and the cross-section illustrating the height changes (b). The thickness distribution (c–g) of the LPE GaSeS flakes according to the series of AFM measurements. Figure S3. Schematic illustration of the PEC photodetectors working in the short current regime. Figure S4. Light sensitive performance of the PEC photodetectors based on GaSeS crystals.
