# Supplementary material for: Growth and Liquid-Phase Exfoliation of GaSe1−xSx Crystals
Source: Materials (Basel). 2022 Oct 12;15(20):7080. doi: 10.3390/ma15207080 (PMC9605154; doi:10.3390/ma15207080)
Supplement: Supplementary file 1 [file materials-15-07080-s001.zip › materials-1906192-supplementary.pdf]

Supporting information

Growth and Liquid-Phase Exfoliation of $GaSe_{1-x}S_x$ crystals

Madi Aitzhanov, Nazim Guseinov, Renata Nemkayeva, Yerulan Sagidolda, Zhandos Tolepov, Oleg Prikhodko and Yerzhan Mukhametkarimov *

National Nanotechnological Laboratory, Al-Farabi Kazakh National University, 71 Al-Farabi Avenue, Almaty 050040, Kazakhstan
* Correspondence: yerzhan.mukhametkarimov@kaznu.kz

1. The determination of the lateral size distribution of the liquid-phase exfoliated (LPE) GaSeS flakes

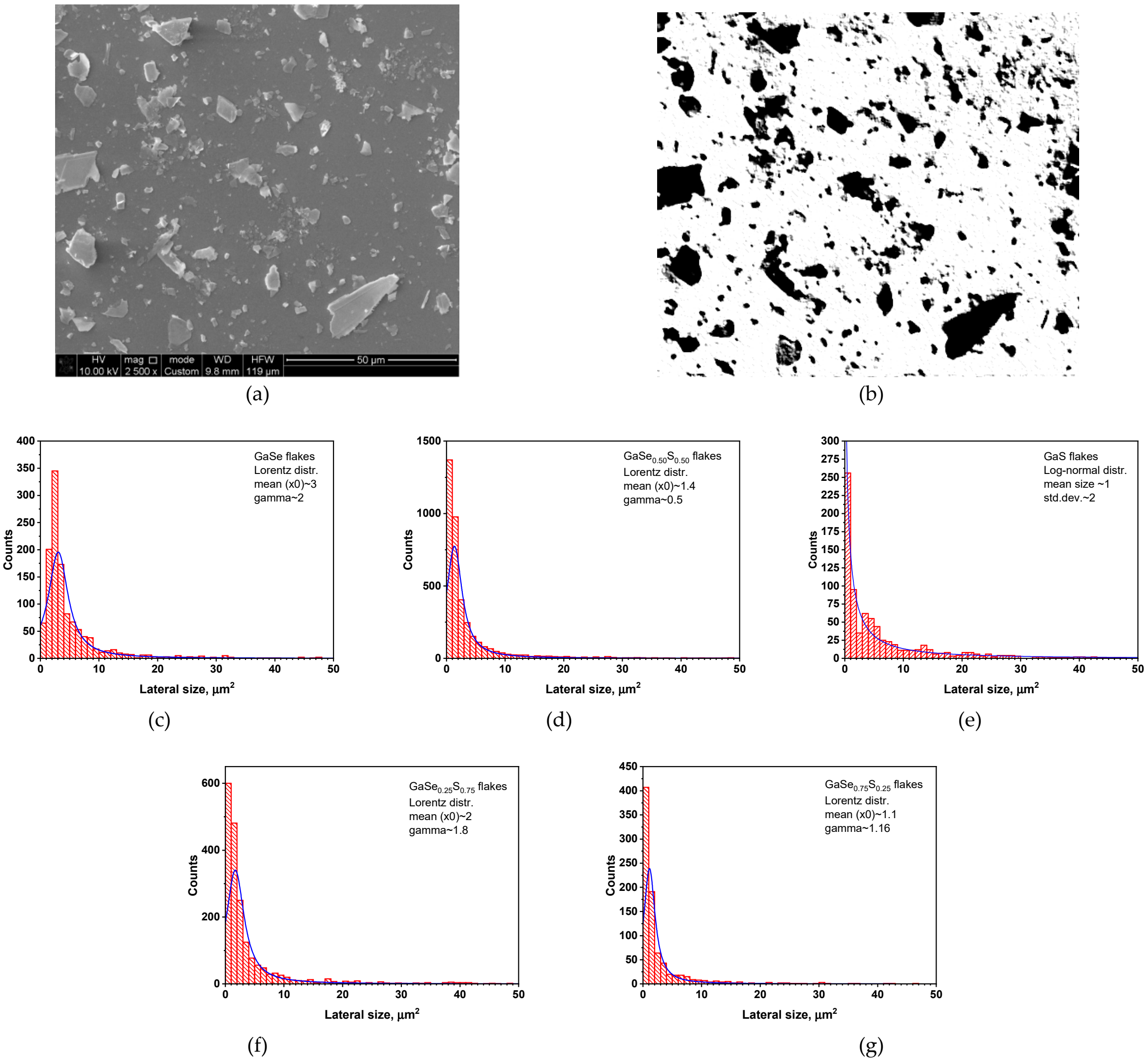

Figure S1. Typical (a) and contrast-adjusted (b) SEM images of the GaSeS flakes (a,b); The lateral size distribution (c–g) of the LPE $GaSe_{1-x}S_x$ flakes. The data of lateral sizes were obtained by analyzing the SEM images (ImageJ software) of the IPA dispersion solution of GaSeS flakes drop-casted onto the m-Si surfaces.

2. The determination of the thicknesses of the GaSeS flakes by atomic force microscopy (AFM)

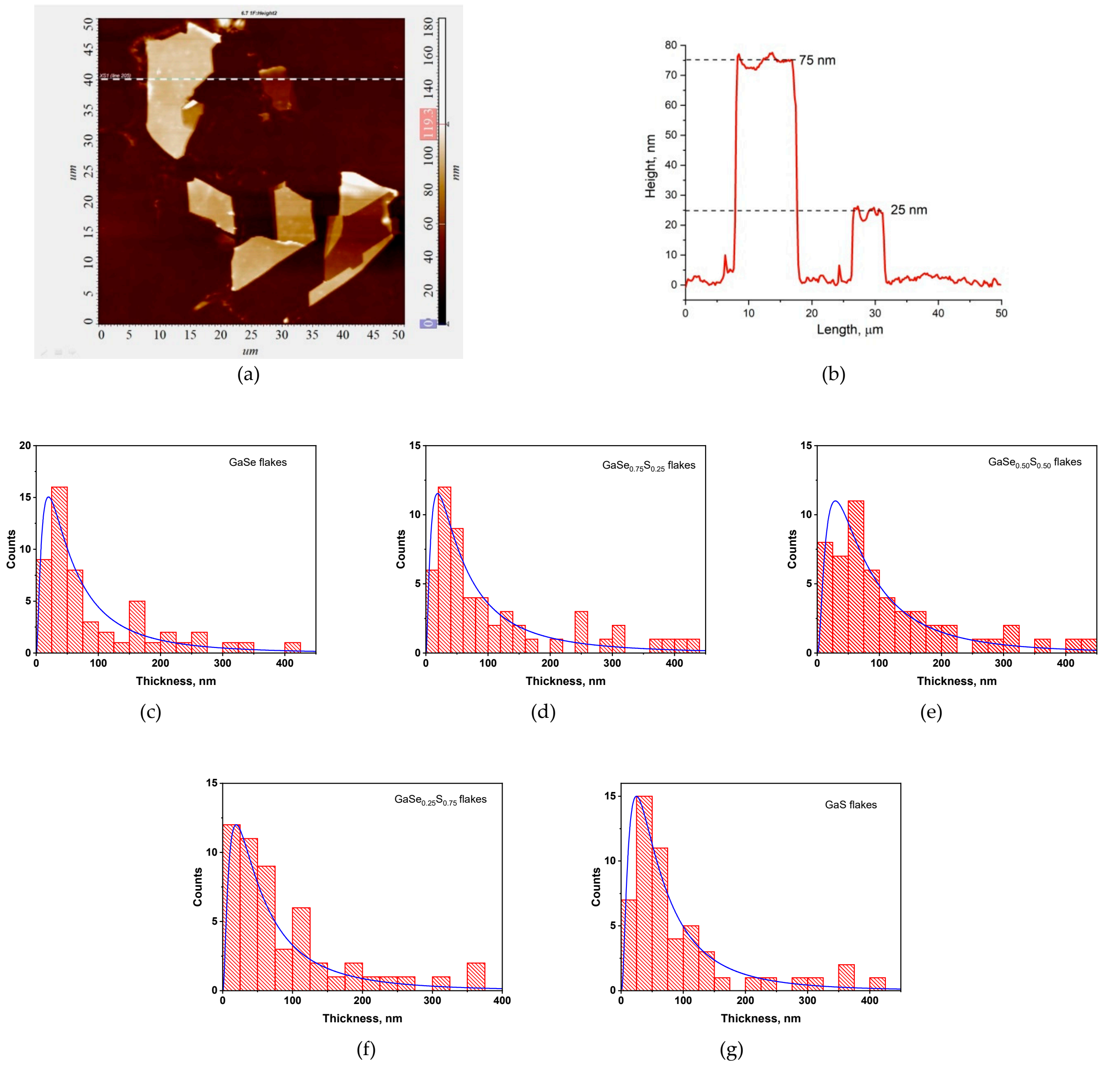

Figure S2. Typical AFM image of the GaSeS flakes (a) and the cross-section (along the white dashed line) illustrating the height changes (b). The thickness distribution (c–g) of the LPE GaSeS flakes according to the series of AFM measurements. Blue lines correspond to the Log-normal distribution (mean~ 4–4.3; std.dev.~0.95–1.1)

3. Photoelectrochemical (PEC) photodetectors based on GaSeS flakes.

To create a working electrode, 1mg of GaSeS flakes were deposited on the surface of 1 cm^2 thin gold film by drop-casting method. Pt mesh was used as a counter electrode. A 0.5 M solution of H_2SO_4 was used as an electrolyte.

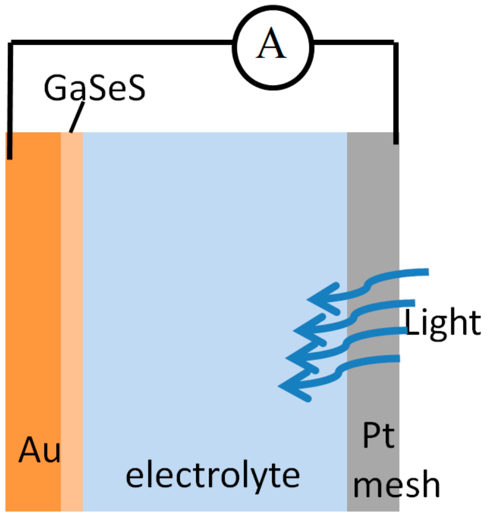

Figure S3. Schematic illustration of the PEC photodetectors working in the short current regime

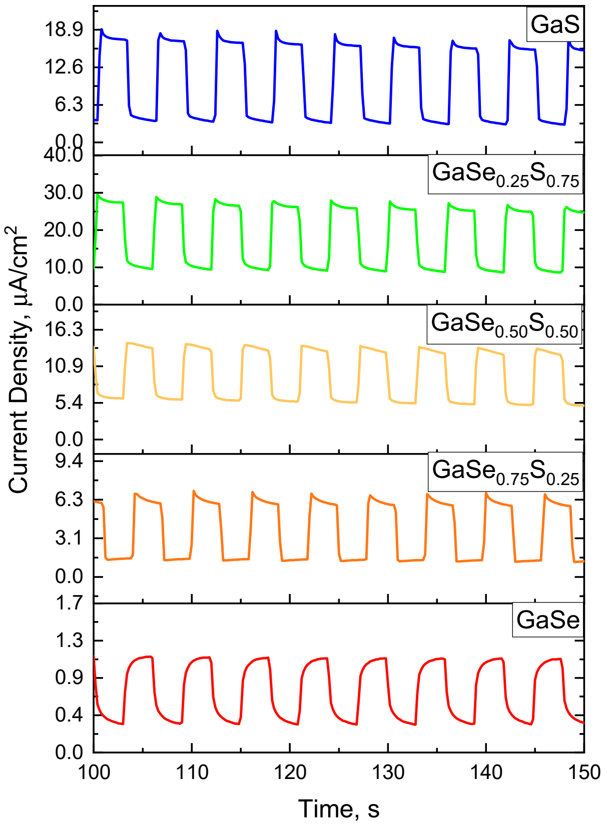

Figure S4. Light sensitive performance of the PEC photodetectors based on GaSeS crystals. The measuring was carried out using modulated illumination of 2mW/cm^2 LED (405 nm) in the short current mode.
